# Plasma Bacterial Metabolites in Crohn’s Disease Pathogenesis and Complications

**DOI:** 10.3390/nu17010074

**Published:** 2024-12-28

**Authors:** Anna Deskur, Filip Ambrożkiewicz, Emilia Samborowska, Wojciech Błogowski, Tadeusz Sulikowski, Andrzej Białek, Iwona Zawada, Krzysztof Dąbkowski, Joanna Mitrus, Jakub Karczmarski, Patrycja Cybula, Agnieszka Paziewska, Teresa Starzyńska

**Affiliations:** 1 Department of Gastroenterology and Hepatology, Pomeranian Medical University in Szczecin, 70-204 Szczecin, Poland; andrzej.bialek@pum.edu.pl (A.B.); iwona.zawada@pum.edu.pl (I.Z.); dabkowskikrzysztof@wp.pl (K.D.); teresa.starzynska@pum.edu.pl (T.S.); 2 Laboratory of Translational Cancer Genomics, Biomedical Center, Faculty of Medicine in Pilsen, Charles University, Alej Svobody 1665/76, 32300 Pilsen, Czech Republic; filip.ambrozkiewicz@lfp.cuni.cz; 3 Mass Spectrometry Laboratory, Institute of Biochemistry and Biophysics, Polish Academy of Sciences, 02-106 Warsaw, Poland; sambor@ibb.waw.pl (E.S.); pocztakuby@gmail.com (J.K.); 4 Institute of Medical Sciences, University of Zielona Góra, ul. Zyty 28, 65-046 Zielona Gora, Poland; w.blogowski@cm.uz.zgora.pl; 5 Department of General, Minimally Invasive, and Gastroenterological Surgery, Pomeranian Medical University in Szczecin, 70-204 Szczecin, Poland; sulikowskit@wp.pl; 6 Institute of Biological Sciences, University of Siedlce, Prusa 14, 08-110 Siedlce, Poland; joanna.mitrus@uws.edu.pl; 7 Institute of Health Sciences, Faculty of Medical and Health Sciences, University of Siedlce, 08-110 Siedlce, Poland; pcybula@ihit.waw.pl; 8 Molecular Biology Laboratory, Department of Diagnostic Hematology, Institute of Hematology and Transfusion Medicine, 02-776 Warsaw, Poland; 9 Warsaw Genomics Inc., 01-682 Warszawa, Poland

**Keywords:** inflammatory bowel diseases, Crohn’s disease, metabolites, SCFA, TMAO, gut microbiome, metabolome

## Abstract

Background/Objectives: Crohn’s disease is known for being associated with an abnormal composition of the bacterial flora, dysbiosis and intestinal function disorders. Metabolites produced by gut microbiota play a pivotal role in the pathogenesis of CD, and the presence of unspecific extraintestinal manifestations. Methods: The aim of this study was a determination of the level of bacterial metabolites in blood plasma in patients with Crohn’s disease. CD patients (29) and healthy individuals (30) were recruited for this study. Bacterial metabolites (SCFAs and TMAO panel) were measured by a liquid chromatography–mass spectrometry system. Results: A significant correlation (*p*-value < 0.05) between CD and bacterial metabolites was obtained for three of eight tested SCFAs; acetic acid (reduced in CD; FC 1.7; AUC = 0.714), butyric acid (increased; FC 0.68; AUC = 0.717), 2MeBA (FC 1.168; AUC = 0.702), and indoxyl (FC 0.624). The concentration of CA (FC 0.82) and choline (FC 0.78) in plasma was significantly disturbed according to the biological treatment. Choline level (FC 1.28) was also significantly disturbed in the patients treated with glucocorticoids. In total, 68.97% of Crohn’s patients presented extraintestinal manifestations (EIMs) of Crohn’s disease, mainly osteoarticular complications. The level of BA was statistically significantly elevated in patients with extraintestinal (FC 0.602) manifestations, while in the group of patients with osteoarticular complications, a significant difference in the level of betaine (FC 1.647) was observed. Conclusions: The analyzed bacterial metabolites of plasma may significantly help in the diagnostic process, and in the monitoring of the disease course and treatment, in a lowly invasive way, as biomarkers after additional research on a larger group of patients.

## 1. Introduction

For many years, studies have been conducted on the possibilities of early diagnosing, monitoring and treatment of unspecific inflammatory bowel diseases (IBDs) such as Crohn’s disease (CD) and ulcerative colitis (UC). It is currently known that an abnormal composition of the bacterial flora—the microbiome—may lead to dysbiosis and intestinal function disorders. Therefore, in the pathogenesis of unspecific inflammatory bowel diseases (CD, UC), and thus in the development of inflammatory lesions, an important role is played by metabolites produced by the gut microbiota [1,2,3,4].

An analysis of the metabolites—metabolomics—is particularly important in chronic gastrointestinal tract inflammation, that is, Crohn’s disease (CD). The etiopathogenesis of CD has not been fully elucidated. CD is frequent in industrialized countries. In recent years, an increase in the incidence has been observed, particularly among individuals aged 15–25 years [5,6]. The development and clinical course of the disease are influenced by immunological, genetic, microbiological and environmental factors. In the pathogenesis, the role of gut microbiota, microbial metabolites produced by it and their interrelations seems of key importance [5,7]. The disease takes a course with exacerbations and remissions, and it is frequently diagnosed late in view of its unspecific manifestations, and test results similar to those observed in patients with other inflammatory intestinal lesions, such as elevated *C*-reactive protein (CRP) concentration. The lesions observed in the disease develop most frequently in the terminal segment of the ileum but can involve any part of the gastrointestinal tract, including the upper part. It is important, thus, to realize that not only the composition of bacteria but also their metabolic activity may be of key importance for understanding the disease and its course. Therefore, great hopes in the diagnosis of the developing lesions and monitoring of the pathogenetic process are raised by an analysis of bacterial metabolites sampled by a non-invasive method, e.g., from plasma or serum, as diagnostic and prognostic biomarkers of Crohn’s disease [5,8,9,10,11].

The aim of the study was an identification of bacterial metabolites, short-chain fatty acids (SCFAs) and TMAO panel, and determination of their levels in venous blood plasma in patients diagnosed with Crohn’s disease. The analyzed metabolites are intermediate or end products of intestinal microbial metabolism, associated with microbial dysbiosis occurring in IBDs. In CD disorders, a great role is played by metabolites derived from short-chain fatty acids (SCFAs): acetate, propionate and butyrate. They exert effects on the development of intestinal inflammatory lesions, immunological reaction and hemostasis [4,12,13,14,15,16,17].

The analyses conducted allowed us to demonstrate that early identification of altered metabolites, their correlation with disease course, location and activity may suggest a risk of development of inflammatory lesions and provide a better and non-invasive monitoring of inflammatory bowel diseases. The determination of the lesions may additionally suggest a progression of the disease, its complications, and may also support a correct treatment of Crohn’s disease.

## 2. Materials and Methods

From 20 May 2022 to 6 February 2023, 29 CD patients (16 women and 13 men) with a mean age of 33.9 years (range of 18–70 years) were diagnosed at the Department of Gastroenterology, Pomeranian Medical University in Szczecin, according to the Porto criteria, modified in accordance with the ECCO guidelines 10. A histological evaluation (including assessment of chronic inflammation, histological activity of inflammation, glandular atrophy, intestinal metaplasia and the presence of granulomas) of diagnostic biopsy specimens was performed by pathologists.

The control group comprised 30 patients (20 women and 10 men) with a mean age of 41.8 years (range—23–76 years). The characteristics of the patients recruited for this study, their clinical information and the characteristics of the controls are shown in Table 1.

This study was performed in accordance with the ethical standards and approval of the local Bioethical committee of the Pomeranian Medical University in Szczecin (No. KB-0012/17/19), and in accordance with the principles of the 1964 Declaration of Helsinki. Informed consent was obtained from all subjects and/or their legal guardian(s).

Inclusion criteria:

- Written informed consent to participate in this study;
- Patients with Crohn’s disease and a control group—adults.

Exclusion criteria:

- Age < 18 years;
- Failure to express informed consent to participate in this research study.

### 2.1. Metabolite (Short-Chain Fatty Acids, TMAO Profile) Profiling

Bacterial metabolites in plasma (SCFAs: acetic acid (AA), butyric acid (BA), caproic acid (CA), isobutyric acid (IBA), lactic acid (LA), 2-methylbutyric acid (2MeBA), Propionic acid (PA), valeric acid (VA)) and TMAO panel (betaine, choline, glycerophosphorylcholine (GPC), indoxyl sulfate, carnitine, trimethylamine (TMA), trimethylamine *N*-oxide (TMAO)) were measured by a liquid chromatography–mass spectrometry system at the Mass Spectrometry Laboratory, Institute of Biochemistry and Biophysics, Polish Academy of Sciences (Warsaw, Poland) and were analyzed with modified protocols described previously by Onyszkiewicz, M., Gawrys-Kopczynska, M., Konopelski, P. et al. Butyric acid, a gut bacteria metabolite, lowers arterial blood pressure via colon–vagus nerve signaling and GPR41/43 receptors, as shown by Pflugers Arch—Eur J Physiol 471, 1441–1453 (2019) [18], Maksymiuk, K.M., Szudzik, M., Gawryś-Kopczyńska, M. et al. Trimethylamine, a gut bacteria metabolite and air pollutant, increases blood pressure and markers of kidney damage including proteinuria and KIM-1 in rats, as shown by J Transl Med 20, 470 (2022) [19].

### 2.2. Statistical Analysis

The data were analyzed by TargetLynx (Waters, Milford, MA, USA) software (https://www.targetlynx.com/). The concentrations of SCFA and TMAO panel metabolites were obtained from a calibration curve. Raw values were log10-transformed. Significant differences were determined by the Wilcoxon test. Benjamini–Hochberg correction for multiple testing was applied. The metabolites were considered significant when FC > 1.5 and *p*-value < 0.05. The associations of the metabolites with clinical and pathological data were assessed by Spearman’s correlation for continuous data or by the Mann–Whitney U test for binary data. A receiver operating characteristic (ROC) analysis was performed to explore the predictive abilities of metabolites, AUC ≥ 0.7 was considered to indicate moderate discriminatory performance. The analysis was performed by R software (v 4.2.2).

The levels of bacterial metabolites were correlated with clinical and histopathological data. Additionally, a follow-up/observation of the patients was carried out, along with an assessment of survival and the course of the disease.

## 3. Results

**Figure 1:**
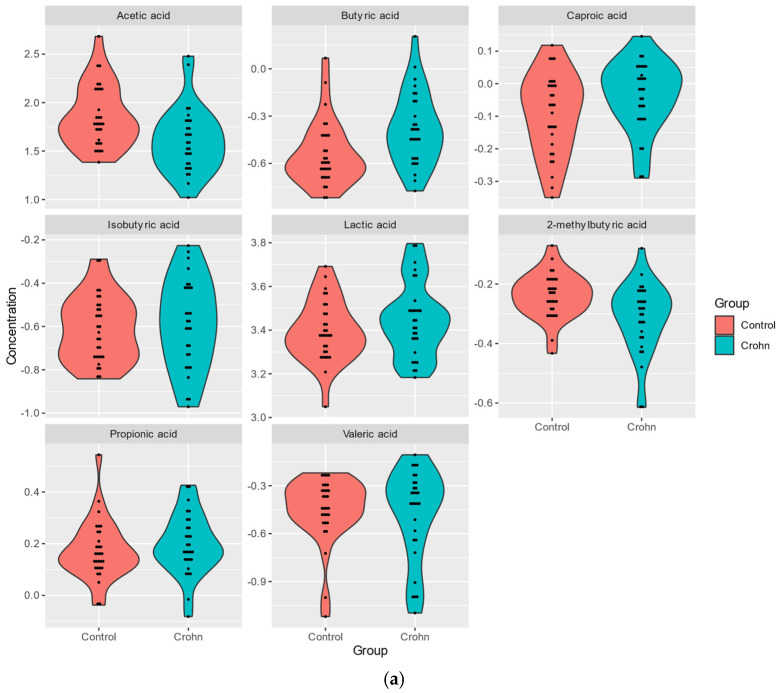

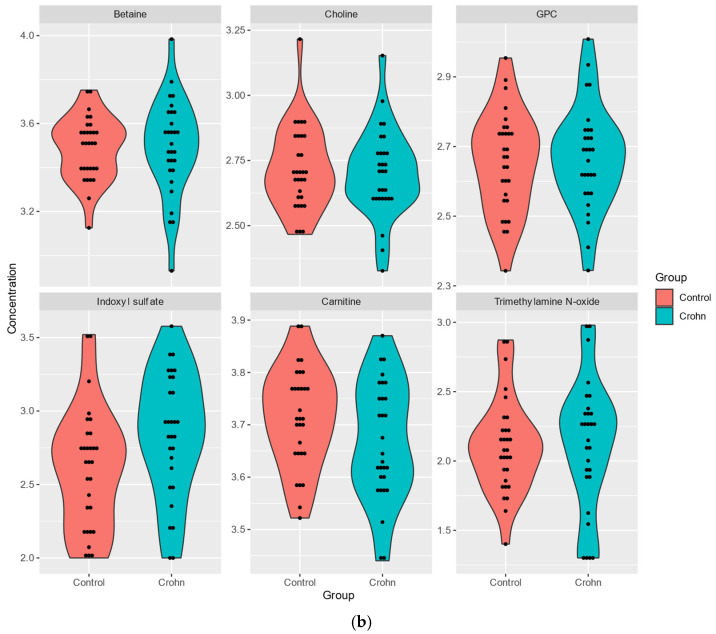
(**a**). Analysis of plasma SCFA metabolites in Crohn’s patients and healthy individuals. (**b**). Analysis of plasma metabolites of TMAO panel in Crohn’s patients and healthy individuals.

The levels of SCFA metabolites and of TMAO panel metabolites were analyzed using mass spectrometry in plasma samples from 29 patients and 30 healthy subjects (Supplementary Table S1). The levels of six out of eight SCFAs detected in plasma samples. BA (FC 0.68), CA (FC 0.89), PA (FC 0.95), LA (FC 0.86), IBA (FC 0.90), VA (FC 0.97), were higher in CD plasma, while those of AA (FC 1.70), 2MeBA (FC 1.17) were reduced (Table 2a, Figure 1a).

The levels of four out of six TMAO detected in plasma samples, indoxyl sulfate (FC 0.62), TMAO (FC 0.82), betaine (FC 0.93), GPC (FC 0.97), were higher in CD plasma, while those of carnitine (FC 1.10), choline (FC 1.03) were reduced (Table 2a,b, Figure 1b).

A significant correlation (*p*-value < 0.05) between CD and bacterial metabolites was obtained only for three of the SCFAs, acetic acid (AA reduced in CD; FC 1.696; *p*-value = 0.004), butyric acid (BA; increased; FC 0.682; *p*-value = 0.004), and 2MeBA (FC 1.168; *p*-value = 0.008), and for indoxyl (FC 0.624; *p*-value = 0.023) of the TMAO panel (Table 2a,b).

**Figure 2:**
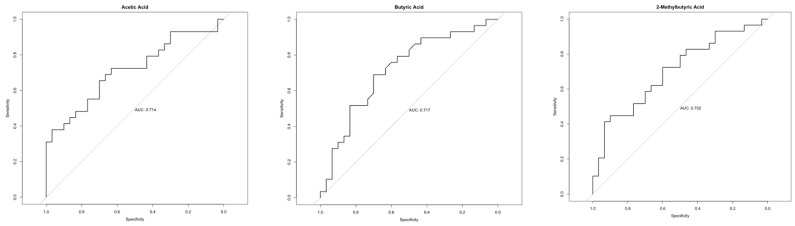
ROC analysis of statistically significantly different metabolites between healthy and Crohn’s patients with AUC > 0.7.

The ROC curves analysis was performed and the area under the curve (AUC) was calculated to determine whether the analyzed metabolites could serve as new diagnostic biomarkers of Crohn’s disease. The highest values (AUC > 0.7; moderate discrimination power) were achieved for SCFAs: AA (AUC = 0.714), BA (AUC = 0.717), and 2MeBA (AUC = 0.702)—Figure 2.

Additionally, betaine (*p*-value = 0.05) and choline (*p*-value = 0.008) were significantly associated with gender (Supplementary Table S2). An oncological family burden was reported for 51.7% of Crohn’s patients. There was no significant correlation between CD, family burden and smoking status (Supplementary Table S3).

### 3.1. Clinical Manifestation of CD with Analysis of Correlation (BMI, Time of Diagnosis, Duration, CRP)

Crohn’s disease was diagnosed in eight patients aged below 16 years (27.6%), in sixteen patients aged from 17 to 40 (55.2%), and in five aged over 40 years (17.2%). Three patients were followed-up and monitored for less than one year 10.3%), five for 1–5 years (17.2%), thirteen for 5–10 years (44,8%), and eight for more than 10 years (27,6%) of disease duration.

**Figure 3:**
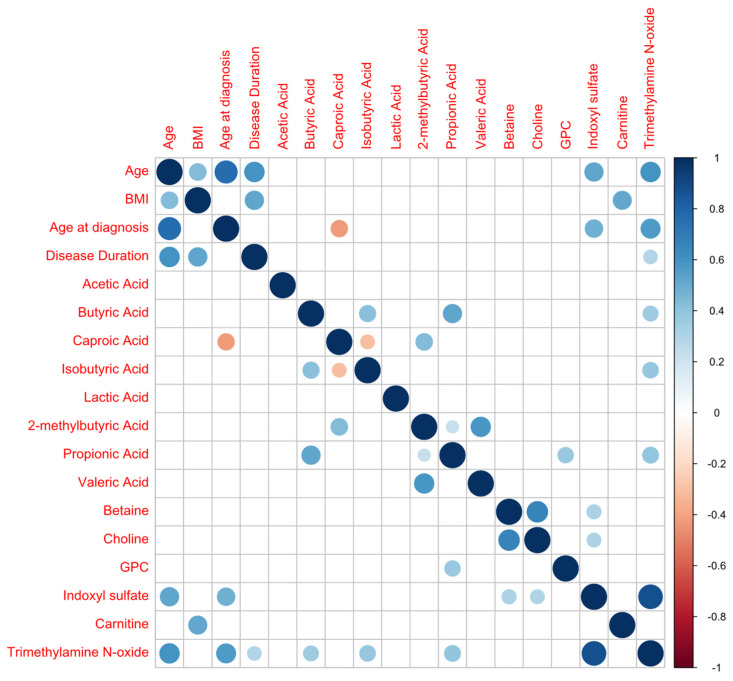
Metabolite correlation analysis (positive correlations are shown in blue; negative correlations in red).

The correlations of the profiles of metabolites with age parameters and BMI are presented in Figure 3, showing the highest correlation between CA, TMAO and indoxyl values and age, and between carnitine and BMI.

Seventeen (58.6%) Crohn’s patients presented remission of CD disease symptoms, and in twelve (41.4%) patients, a mild course of the disease was observed. According to CDAI, only the concentration of TMAO significantly (*p*-value < 0.05) differed between patients with an FC of 0.35, and *p*-value = 0.0456. Additionally, indoxyl (*p*-value = 0.044) was correlated with the location of Crohn’s lesions.

Crohn’s disease patients tested for the presence of *C*-reactive protein (CRP) in blood plasma showed no significant increase in the concentration of bacterial metabolites.

#### 3.1.1. Inflammation Status

**Figure 4:**
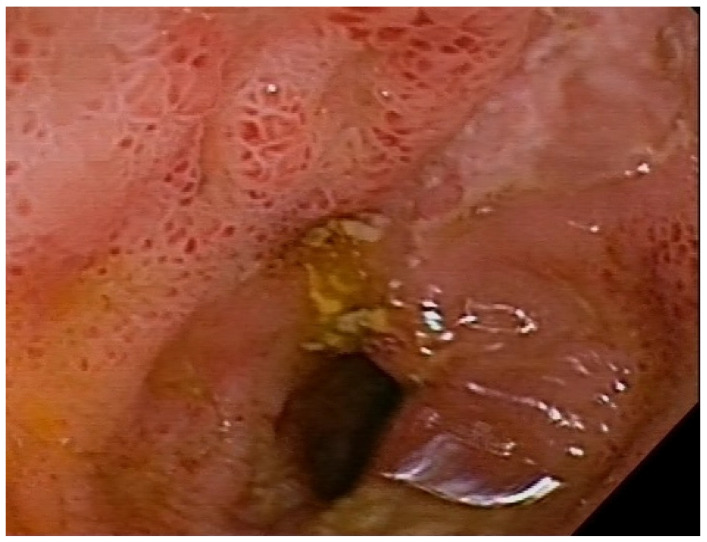
Mucositis in the distal segment of the ileum, with ulcers in a patient with Crohn’s disease.

**Figure 5:**
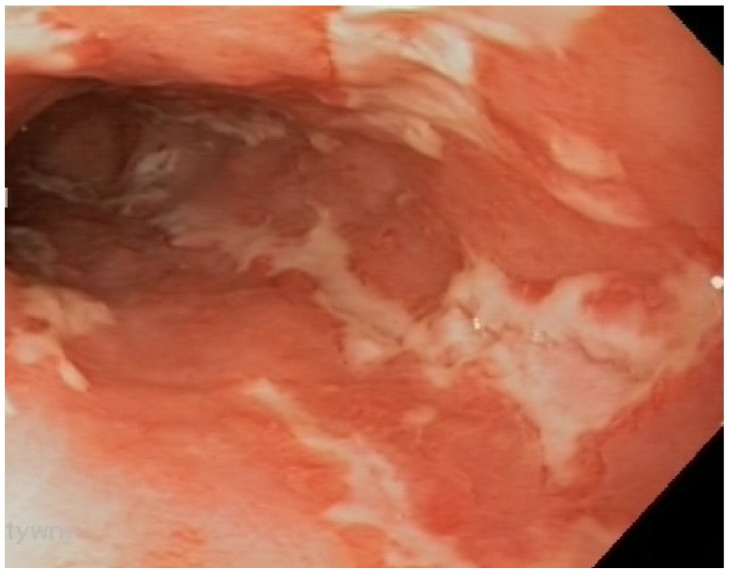
Ulcerative mucositis in the large intestine of a patient with Crohn’s disease.

**Figure 6:**
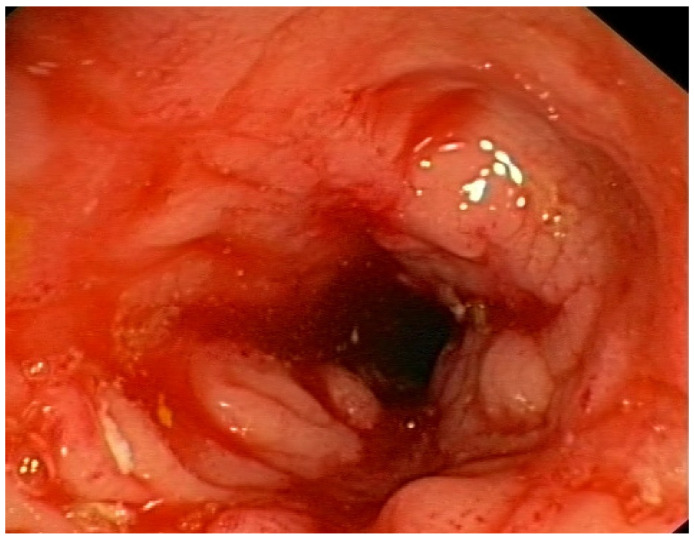
Inflammation, swelling and narrowing of the intestinal lumen in a patient with Crohn’s disease.

Thirteen (44.8%) CD patients presented inflammatory status of Crohn’s disease (Figure 4, Figure 5 and Figure 6), and in sixteen patients, the disease showed a structuring behavior. The bacterial metabolites showing a significant increase in blood plasma concentration according to this behavior classification included LA (*p*-value = 0.0251; FC 0.753) and indoxyl (*p*-value = 0.0484; FC 0.515)—Table 3.

Among the Crohn’s patients, five were infected with *Helicobacter pylori* (HP) with significant differences observed for indoxyl sulfate (*p*-value = 0.0423; FC 0.578) and TMAO (*p*-value 0.0227; FC 0.286) in the concentrations in blood plasma samples – Table 4.

In 16 (55.2%) patients, an inflammation of the upper gastrointestinal tract segment was present (esophagus—3, stomach—14, duodenum—11), associated with a significant increase in indoxyl (*p*-value = 0.0168; FC 0.514) and TMAO (*p*-value = 0.02; FC 0.370) in plasma samples. Eleven patients presented duodenitis and a significant increase in blood plasma concentration of GPC (*p*-value = 0.0214; FC 0.727, TMAO (*p*-value = 0.023; FC 0.5195), carnitine (*p*-value = 0.035; FC 0.827) and indoxyl (*p*-value = 0.0455; FC 0.539)—Table 5.

#### 3.1.2. Crohn’s Disease Complications

Typical perianal complications were observed in 15 patients. In 20 (68.97%) Crohn’s patients, extraintestinal manifestations (EIMs) of Crohn’s disease, mainly osteoarticular complications, were present (Table 6). They were mainly related to bones and joints (peripheral arthralgia, spondylalgia, ankylosing spondylitis) and were present in fifteen patients (51.7%), but they also included conjunctivitis in one patient (3.4%), skin manifestations in three patients (10.3%), aphthous ulcers in the oral cavity in four patients (13.8%), hepatic complications (hepatic steatosis in four patients, 13.8%), biliary tract complications (PSC—primary sclerosing cholangitis in two patients, 6.9%), nephrolithiasis in one patient (3.4%), and cholelithiasis in three patients (10.3%).

**Figure 7:**
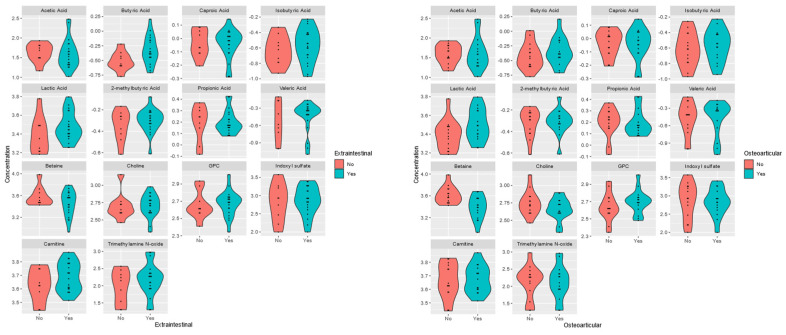
Violin plots for the analyzed plasma metabolites’ differences between plasma patients CD with (yes) extraintestinal and osteoarticular complications and without (no).

The level of BA was statistically significantly elevated in patients with extraintestinal (*p*-value = 0.044; FC 0.602) and perianal (*p*-value = 0.041) manifestations, while in the group of patients with osteoarticular complications, a significant difference in the level of betaine (*p*-value = 0.002; FC 1.647) was observed—Table 6, Figure 7.

### 3.2. Treatment

Treatment decisions were made depending on the severity and progression of the disease and its location, to reduce complications and inflammation, and to obtain clinical remission of the disease. Table 7 presents Crohn’s therapy administered in the patients; 14.48% of the patients with Crohn’s disease were subjected to the present biological treatment, which resulted in significant changes in the concentration of CA (at present: *p*-value = 0.014; FC 0.82, and after the biological treatment in the past: CA *p*-value = 0.0196), and choline (*p*-value = 0.033; FC 0.78) in blood plasma. The choline level (*p*-value = 0.049; FC 1.28) was also significantly disturbed in the patients (9; 31.03%) treated with glucocorticoids.

Sixteen (55.17%) patients with Crohn’s disease were treated with immunosuppressive drugs, which did not result in significant statistical differences in the profile of metabolites in blood plasma, similarly to ASA therapy (26 patients; 89.65% with Crohn’s disease).

## 4. Discussion

Metabolomics is a dynamically developing science, increasingly frequently used in medical diagnostic procedures. Based on the changes in the metabolic profile of human body fluids, we look for specific biomarkers appearing already at the onset of a disease.

Bacterial metabolites produced by the gut microbiota exert a great influence on the development of unspecific inflammatory bowel diseases (IBDs) such as Crohn’s disease (CD) and ulcerative colitis (UC) [5,20]. Of great importance for making the diagnosis of CD may be the tests for changes in the functioning of intestinal micro-organisms, assessed through changes in the levels of TMAO and SCFA metabolites. These tests determine the relationship between the metabolism of intestinal micro-organisms and the patient’s disease status, and provide a better insight into the interactions between the host and bacterial flora in IBD [21,22].

Our study has demonstrated that identification of the changes in the levels of metabolites in plasma of patients diagnosed with Crohn’s disease enables a determination of the risk associated with intestinal inflammatory conditions and their better monitoring. A particular role is played by the short-chain fatty acid (SCFA) metabolites analyzed in this study, since an abnormal function of the intestinal barrier causes an increased penetration of SCFA metabolites from the intestine into blood—the so-called “leaky gut” syndrome, which has been confirmed in our studies. That finding may help in the diagnosis and monitoring of the treatment of CD, while the disturbed balance of the SCFAs produced by intestinal bacteria may serve as a marker of inflammatory bowel diseases [21]. SCFAs as products of bacterial metabolism are responsible for the maintenance of homeostasis in the human gastrointestinal tract [6,11]. Intestinal flora participates in the host’s metabolism, producing great amounts of small molecules and hormones, and the metabolites enter the host’s body to participate in systemic circulation and to influence the homeostasis of the body [23]. Metabolome variability is associated with differences in the microbiome between healthy individuals and patients—dysbiosis [24,25,26,27]. The relationship between intestinal dysbiosis and the SCFA level in intestinal inflammatory response is a complex process [26]. Inflammatory reactions accompanying IBDs, including CD, are characterized by evident changes concerning the attainment of metabolic energy of the microflora and of the host.

In healthy mucosa, energy requirements are high to maintain the normal complex of epithelial cells and ensure their proper functioning. Energy comes from diet and microflora. The metabolites of microbial origin (SCFAs), including butyrate, are substrates for ATP production, which ensures intestinal homeostasis. On the other hand, in IBDs, as a result of dysbiosis and disturbed absorption of nutrients, the “hungry gut” is characterized by excessive energy demand of the mucosa, particularly in the case of an active inflammatory condition [28]. Moreover, in patients with an IBD, the mitochondria, as energy-providing centers located in the epithelial cells, are deformed, which may also lead to disorders in energy attainment [29].

A dysbiosis of the gut microbiota (GM) causes an impairment of the communication between GMO (gut microbiota ontology) and immune cells. Furthermore, recent studies have demonstrated the key role of bacterial post-biotics (metabolites) in organizing the immune response of the host [30]. The fact that bacterial metabolites constitute a type of link between the microbiome and immune system is also important. It has been proven that butyrate exerts anti-inflammatory effects both on immune cells and intestinal epithelial cells (mucus-secreting goblet cells, absorptive enterocytes, hormone-producing enteroendocrine cells, lectin-secreting Paneth cells and antimicrobial peptides). It is suggested that SCFAs exert anti-inflammatory effects in the intestinal mucosa since they are responsible for the inhibition of the expression of adhesive molecules formed as a result of the on-going inflammatory process, and finally, for the inhibition of the adhesion of monocytes/macrophages and neutrophils [30]. It has been also demonstrated that butyrate and other SCFAs are activators of membrane G-protein-coupled receptors (GPRs) responsible for intracellular information flow. The fatty-acid-induced activation of membrane receptors—GPR43 (FFAR2, FFAR3), GPR109A and GPR164—results in the production and secretion of interleukin-10 (IL-10) and IL-18 in order to alleviate the inflammatory condition [28].

It has been also proven that GMO metabolites have an ability to migrate to various host’s organs, e.g., to the central nervous system, where they can participate in the regulation of immune responses [31].

A significant increase in the butyric acid (BA; FC = 0.682) level has been demonstrated. According to Säemann et al. [32], butyric acid has the highest potential of inhibition of histone deacetylase (HDAC), which leads to the induction of T-regulator (T-reg) cells and, because of their increased proliferation, the excessive immune reaction is silenced [30]. It seems that butyric acid, as one of the metabolites analyzed in the studies conducted, may be a new biomarker, BA (AUC = 0.717), determining the pathological changes beginning in the body. A similar suggestion has been proposed by Xu et al. (2022) [8], who suggested that SCFAs produced by intestinal bacteria may serve as markers of inflammatory bowel diseases.

As a result of the pathogenic process in CD, body homeostasis is disturbed. Dynamic quantitative and qualitative changes then occur in the metabolome of body fluids. The search for those discreet changes in plasma enabled us to identify not only the potential biomarkers of the disease but also metabolites, which correlated with the patients’ clinical condition, CDAI; TMAO (*p*-value = 0.0456), location of Crohn’s lesions; indoxyl (*p*-value = 0.044) or classification (inflammatory status/stenosing form of the disease); LA (*p*-value = 0.0251); and indoxyl (*p*-value = 0.048).

That is of significant importance since the course of the disease with intestinal stenosis is associated with a risk of surgical intervention and necessity of treatment intensification. The CDAI scale considers a number of significant factors such as the presence of signs of clinical exacerbation of the disease and the presence of extraintestinal manifestations or laboratory parameters (hematocrit), and it is commonly used by clinicians for the assessment of the condition of CD patients and for making therapeutic decisions. Our report presents the potential of selected markers in the clinical assessment of patients with disease exacerbation, which is important due to the limitations of available markers. The utilized serum markers of the disease (CRP) are characterized by a lack of specificity or false negative results in some forms of the disease (only limited to the small intestine) and in liver diseases [33]. The concentrations of serum markers and of the commonly used marker determined in feces (calprotectin), similarly to bacterial profiles and products of the bacteria—metabolites—change due to a number of factors, including diet and treatment. The metabolome of CD patients can be stabilized, e.g., through exclusive enteral nutrition (EEN), personalized diet (CD-TREAT) based on nutrients of composition similar to EEN, or an elimination diet (CDED) combined with a partial enteral nutrition (PEN). These diet therapies induce CD remission, contributing to changes in the microbiome, which lead to a reduction in the intestinal inflammatory condition and, in consequence, to an improvement in the patient’s clinical condition [24,27]. Together with the changes in short-chain fatty acids (a reduction in acetate and butyrate), clinical improvement was observed in 80%, and remission in 60% of cases [24]. Remission also induced by CDED+PEN, and EEN was associated with significant changes in the metabolites associated with inflammatory bowel disease [34].

Changes in the composition of the microflora and SCFA profile (e.g., increased butyrate concentration in feces) were also visible after fecal microbiota transplantation (FMT) [35]. A clinical response was obtained in 75% and clinical remission in 25% of patients with CD. In addition to that, in CD patients, a decrease was demonstrated in Blautia, Dorea and Eubacterium before FMT and an increase in Collinsella, Dorea and Eubacterium was found after FMT [35].

In children with an IBD, significant differences were described in the BA level compared with the control group and they depended on the effectiveness of the nutritional therapy—exclusively enteral nutrition and administration of infliximab [22]. A therapy with TNF inhibitors and administration of immunomodulatory drugs [28,30] may result in SCFA changes in feces (increased isobutyric acid concentration) [36].

Elevated propionic acid concentrations were observed in patients with IBDs treated with trimebutine (*p*-value = 0.031) and higher isobutyric acid concentrations were found in patients treated with biological drugs compared with untreated patients (*p*-value = 0.014) [36].

In our current study, significant changes were observed in caprylic acid (CA) concentration (FC 0.82; *p*-value = 0.014) and choline level (FC 0.78; *p*-value = 0.033) in the plasma of patients with CD receiving biological treatment (14 individuals). Significant changes in choline content (FC 1.28; *p*-value = 0.049) were also obtained in patients treated with glucocorticoids. However, similarly to the case of ASA therapy administration, no statistically significant differences were observed in the profile of metabolites in patients receiving immunosuppressive treatment.

Crohn’s disease (CD) is a chronic systemic disease with gastrointestinal symptoms and signs. It is an inflammatory bowel disease (IBD) of immune origin. It may not only involve a segment of the gastrointestinal tract (GIT) but also the osteoarticular system, liver, bile ducts, skin and eyes. The pathogenesis of CD and its extraintestinal complications is, however, still not sufficiently elucidated. Nevertheless, it is supposed that the mucosa of the gastrointestinal tract in CD also affects the immune response in extraintestinal locations [30,37].

Extraintestinal manifestations occur with various frequency (5–50% of IBD patients), severity and course. Isolated or multiple disorders and pathological lesions may develop before or after intestinal manifestations or after making the diagnosis. Extraintestinal complications such as aphthae, arthritis, erythema nodosum, peripheral arthritis, and episcleritis may be associated with intestinal activity and exacerbation of gastric problems or, e.g., ankylosing spondylitis, uveitis and primary sclerosing cholangitis, may be independent of the activity or severity of intestinal diseases [37]. An IBD also predisposes patients to bone metabolic disorders leading to low bone mass (14–42% of patients). In this study conducted in patients with Crohn’s disease, the most frequent extraintestinal manifestations were osteoarticular complications: peripheral arthralgia, spinalgia, ankylosing spondylitis (51.7%). In this group of patients, a significant difference in betaine level was obtained (*p*-value = 0.002; FC 1.647).

The significantly lower level of betaine in patients with CD may be, similarly to the case of IBDs, correlated with a gut inflammatory condition. It has been demonstrated that the compound has anti-inflammatory properties and can alleviate colitis [38]. Experiments on mice with IBDs have proven that betaine is an inhibitor of proinflammatory cytokines (IL-1β, IL-6 and TNFα); it strengthens the intestinal barrier and changes the gut microflora [39], leading to the possibility of using new drugs for the treatment of human diseases [39].

The chronic inflammatory character of CD therefore requires a constant monitoring of disease activity and a targeted effective treatment that alleviates the pathological processes, and minimizes inflammatory conditions and adverse effects but also favorably affects the bacterial composition, i.e., the microbiome and metabolome of patients. As we have proven in our studies, composition can change not only due to the disease but also due to the treatment administered. The extraintestinal complications exert a negative effect on the pathological process and the quality of life of patients with IBDs. Therefore, it is important to study the effect of CD on the metabolome through an assessment of the levels of TMAO and SCFA metabolites and to determine the relationship between intestinal metabolism and a patient’s disease status in accordance with the previously presented knowledge that the microbiota of CD patients differs from that of healthy individuals [23,40].

Intestinal dysbiosis plays a key role in the development of IBDs, including CD. The role of bacterial metabolites, such as SCFAs—succinic acid and lactic acids—and the role of environmental factors in their production in the course of IBDs remain unclear.

A limitation of this study is the small number of patients recruited for this project. This preliminary study will be continued with larger groups of patients to discover and analyze plasma metabolites that will provide the highest specificity and sensitivity for the diagnosis of CD patients. By monitoring the metabolites’ concentration, and following up with the patients, their condition, and their diet, we will gain information about the progression of the disease, complications, and the patients’ outcomes [41,42,43,44].

## 5. Conclusions

As part of realization of this project, the knowledge of the pathogenesis of Crohn’s disease has been extended. It has been confirmed that Crohn’s disease is associated with dysbiosis and with changes in the profiles of bacterial metabolites in plasma. The pathological process accompanied by dysbiosis together with systemic manifestations of CD requires sensitive and specific diagnostic methods and possibilities of constant monitoring in order to improve the patients’ health conditions, to prevent disease progression and to reduce mortality. The bacterial metabolites analyzed by us may significantly help in the diagnostic process, selection of effective therapy and monitoring of disease course and treatment in an easily available, rapid and lowly invasive way. The molecules identified may be used in future studies as potential diagnostic or prognostic biomarkers of Crohn’s disease. Some of the metabolites identified correlate with disease severity and may serve as markers of a severe disease course. A better knowledge of the plasma metabolomics of patients with CD and identification of the biomarkers may contribute to earlier diagnosis and improvement of the treatment of IBD [41].

## Figures and Tables

**Table 1:** Characteristics of Crohn’s patients and healthy controls. NA: not available.

	Crohn’s Disease	Control
Number	29	30
Sex (male/female)	13/16	10/20
Age	33.7 ± 14	41.8 ± 14
BMI—body mass index [kg/m^2^]	23.4 ± 4.9	31.7 ± 6.5
WBC—white blood cell [tys/µL]	7.8 ± 2.8	6.5 ± 1.2
RBC—red blood cell [mln/µL]	4.5 ± 0.7	4.8 ± 0.5
HGB hemoglobin [g/dL]	12.6 ± 1.9	14.3 ± 1.3
HCT—hematocrit [%]	37.6 ± 4.9	41.4 ± 3.3
PLT platelet count [tys/µL]	289.1 ± 91.2	276.2 ± 48.4
MCV mean corpuscular volume [fL]	84.1 ± 7.7	85.5 ± 4.7
ALT—alanine aminotransferase [U/L]	19.0 ± 8.6	NA
AST—aspartate aminotransferase [U/L]	22.1 ± 6.9	NA
GGTP—gamma-glutamyl transferase [U/L]	16.7 ± 10.6	NA
Creatinine [mg/dL]	0.8 ± 0.1	0.8 ± 0.1
CRP—*C*-Reactive Protein [mg/L]	15.4 ± 21.1	NA
Remission, *n* [%]	16 [55.2]	-
Family burden, *n* [%]	15 [51.7]	14 [46.7]
Age of diagnosis, *n* [%]		
≤16	8 [27.6]	-
17–40	16 [55.2]	-
40	5 [17.2]	-
Disease duration [years]		
≤1	3 [10.3]	
1–5	5 [17.2]	
5–10	13 [44.8]	
>10	8 [27.6]	
Operations	16 [55.2]	
Treatment (current):		
Immunosuppressants-	16 [55.17]	
Glucocorticoids-	9 [31.03]	
Biological therapy-	14 [48.28]	
Aminosalicylic acid (ASA)	26 [89.66]	
Inflammation of upper digestive tract (esophagus, stomach, duodenum)	16 [55.2]	
Location, *n* [%]		
Ileal	4 [13.8]	-
Colonic	5 [17.2]	-
Ileocolonic	20 [69.0]	-
*H. pylori* infection, *n* [%]	8 [27.6]	-
Smoking status, *n* [%]		
Current-smoker	5 [17.2]	0
Ex-smoker	4 [13.8]	1 [3.0]
Never-smoker	20 [68.9]	29 [97.0]

**Table 2:** (**a**) Analysis of plasma SCFA metabolites in Crohn’s patients and healthy individuals. (**b**) Analysis of plasma metabolites of TMAO panel in Crohn’s patients and healthy individuals.

**(a)**		
***SCFA Metabolites***	***p-Value***	***FC Control/Crohn’s***
**AA**	***0.004***	**1.696**
**BA**	***0.004***	**0.682**
**2MeBA**	***0.008***	**1.168**
CA	*0.080*	0.892
PA	*0.262*	0.948
LA	*0.294*	0.863
IBA	*0.510*	0.905
VA	*0.722*	0.973
**(b)**		
***Metabolite of TMAO Panel***	***p-Value***	***FC Control/Crohn’s***
**Indoxyl sulfate**	***0.023***	**0.624**
carnitine	*0.121*	1.103
TMAO	*0.539*	0.816
betaine	*0.679*	0.926
GPC	*0.863*	0.966
choline	*0.958*	1.033

**Table 3:** Analysis of plasma metabolites of Crohn’s patients and healthy individuals, according to disease behavior.

Disease Behavior Structuring/Inflammatory	*p*-Value	FC
AA	*0.983*	0.645
BA	*0.199*	0.690
betaine	*0.308*	1.028
CA	*0.199*	1.107
choline	*0.308*	0.789
GPC	*0.812*	0.948
IBA	*0.268*	0.840
**Indoxyl sulfate**	***0.0484***	**0.515**
carnitine	*0.268*	0.890
**LA**	***0.0251***	**0.753**
2MeBA	*0.88*	0.987
PA	*0.589*	0.913
TMAO	*0.0829*	0.423
VA	*0.742*	0.966

**Table 4:** Analysis of plasma metabolites of Crohn’s patients and healthy individuals, according to HP infection.

Metabolite	*p*-Value	FC
AA	*0.668*	1.730
BA	*0.071*	0.489
betaine	*0.717*	0.906
CA	*0.371*	1.121
choline	*0.717*	1.029
GPC	*0.0832*	1.439
IBA	*0.272*	0.764
**Indoxyl sulfate**	***0.0423***	**0.578**
carnitine	*0.818*	1.003
LA	*0.192*	1.219
2MeBA	*0.272*	1.127
PA	0.371	0.863
**TMAO**	**0.0227**	**0.286**
VA	1	0.958

**Table 5:** Analysis of metabolites in the plasma of Crohn’s patients and healthy individuals, depending on inflammation of the upper digestive tract segment—duodenum.

Inflammation of Upper Digestive Tract	*p*-Value	FC	Inflammation of Upper Digestive Tract—Duodenum	*p*-Value	FC
AA	*0.199*	0.843	AA	*0.084*	1.122
BA	*0.249*	0.776	BA	*0.492*	0.846
betaine	*0.983*	0.916	betaine	*0.947*	0.922
CA	*0.170*	1.110	CA	*0.642*	1.017
choline	*0.232*	0.766	choline	*0.438*	0.825
GPC	*0.714*	0.898	**GPC**	***0.021***	**0.727**
IBA	*0.779*	0.972	IBA	*0.340*	1.235
**Indoxyl sulfate**	***0.017***	**0.514**	**Indoxyl sulfate**	***0.046***	**0.539**
carnitine	*0.215*	0.873	**carnitine**	***0.035***	**0.827**
LA	*0.199*	0.765	LA	*0.122*	0.733
2MeBA	*0.650*	0.907	2MeBA	*0.492*	0.895
PA	*0.056*	0.801	PA	*0.055*	0.813
**TMAO**	***0.020***	**0.370**	**TMAO**	***0.023***	**0.519**
VA	*0.554*	0.956	VA	*0.637*	1.185

**Table 6:** Crohn’s disease manifestations (complications) in patients, and correlation with plasma metabolites.

*Complications/Manifestations of CD*	*Perianal (n = 15)*		*Extraintestinal (n = 20)*		*Osteoarticular (n = 15)*	
*Metabolite*	*p-Value*	*FC*	*p-Value*	*FC No/Yes*	*p-Value*	*FC No/Yes*
AA	*0.112*	1.17	*0.532*	0.778	*0.847*	0.647
BA	***0.041***	0.64	***0.044***	**0.602**	*0.425*	0.822
betaine	*0.683*	1.15	*0.253*	1.323	***0.002***	**1.647**
CA	*0.847*	1.04	*0.945*	1.007	*1.000*	1.003
carnitine	*0.134*	0.85	*0.317*	0.873	*0.847*	0.990
choline	*0.561*	1.00	*0.417*	1.033	*0.400*	1.238
GPC	*0.983*	0.98	*0.501*	0.969	*0.331*	0.885
IBA	*0.914*	1.04	*0.501*	0.839	*0.591*	0.895
indoxyl	*0.879*	1.11	*0.962*	1.126	*0.631*	1.290
LA	*0.112*	0.83	*0.183*	0.842	*0.093*	0.777
2MeBA	*0.310*	0.93	*0.532*	0.917	*0.880*	0.981
PA	*0.270*	0.90	*0.627*	1.008	*0.505*	1.006
TMAO	*0.444*	0.72	*0.383*	0.585	*0.878*	0.956
VA		0.88	*0.637*	0.940	*0.600*	0.982

**Table 7:** Therapy of Crohn’s patients and plasma metabolite profiles correlated with the type of treatment.

	*Biological Therapy; n = 14*		*Glucocorticoid Therapy; n = 9*		*Aminosalicylates; n = 26*		*Immunosuppressants* *;* *n = 16*	
*Metabolite*	*p-Value*	FC	*p-Value*	FC	*p-Value*	FC	*p-Value*	FC
AA	*0.652*	1.00	*0.835*	1.50	*0.660*	2.52	*0.746*	1.01
BA	*0.652*	0.86	*0.764*	0.80	*0.350*	0.64	*0.374*	1.45
betaine	*0.377*	0.80	*0.562*	1.12	*0.281*	0.72	*0.914*	0.95
CA	***0.014***	**0.82**	*0.501*	1.08	*0.710*	1.05	*0.846*	1.01
choline	***0.033***	**0.78**	***0.049***	**1.28**	*0.350*	0.75	*0.948*	0.98
GPC	*0.217*	0.82	*0.444*	1.16	*0.067*	0.68	*0.475*	0.93
IBA	*0.377*	1.19	*0.472*	0.89	*0.660*	0.88	*0.503*	1.20
Indoxyl sulfate	*0.810*	0.83	*0.099*	0.64	*0.452*	1.24	*0.497*	0.75
carnitine	*0.949*	1.00	*0.234*	1.12	*0.429*	0.84	*0.682*	0.95
LA	*1.000*	1.02	*0.085*	1.22	*0.516*	0.78	*0.101*	1.24
2MeBA	*0.112*	0.87	*0.116*	1.16	*0.761*	1.03	*0.092*	0.87
PA	*0.914*	0.97	*0.764*	0.99	*0.281*	0.81	*0.423*	1.09
TMAO	*0.844*	1.15	*0.085*	0.36	*0.886*	0.78	*0.645*	1.23
VA	*0.793*	1.02	*0.524*	1.13	*0.197*	1.50	*0.693*	0.91

## Data Availability

All data are available in the Supplementary Materials—tables (available online).

## Supplementary Materials

The following supporting information can be downloaded at: https://www.mdpi.com/article/10.3390/nu17010074/s1, Table S1a. Metabolites (SCFA) concentrations [µM] for plasma samples. Table S1b. Metabolites (TMAO panel) concentrations [µM] for plasma samples. Table S2. concentrations [µM] for plasma samples according to gender. Table S3a. Metabolites concentrations [µM] for plasma samples according to family burden (oncological). Table S3b. Metabolites concentrations [µM] for plasma samples according to family burden (NCHZJ).

## Abbreviations

IBDs	inflammatory bowel diseases
CD	Crohn’s disease
UC	ulcerative colitis
AA	acetic acid
BA	butyric acid
CA	caproic acid
IBA	isobutyric acid
LA	lactic acid
2MeBA	2-methylbutyric acid
PA	propionic acid
VA	valeric acid
GPC	glycerophosphorylcholine
TMA	trimethylamine
TMAO	trimethylamine *N*-oxide
ROC	receiver operating characteristic
GM	gut microbiota
GPRs	G-protein-coupled receptors
HDAC	histone deacetylase
EEN	exclusive enteral nutrition
CDED	CD elimination diet
PEN	partial enteral nutrition
